# Structural modeling supports an interaction between the *Drosophila* Estrogen-Related Receptor and Sima/HIF1α

**DOI:** 10.17912/micropub.biology.002303

**Published:** 2026-10-26

**Authors:** Yuan Feng, Jason M Tennessen

**Affiliations:** 1 Indiana University, Bloomington, IN, United States

## Abstract

The *Drosophila* Estrogen-Related Receptor (dERR) is an orphan nuclear receptor that regulates developmental metabolism, yet the protein cofactors that modulate its activity remain poorly defined. Here, we used the AI-based FlyPredictome platform to identify candidate dERR interaction partners and evaluate their predicted structural interfaces. Among the highest-confidence interactors is the transcription factor Sima, which represents the *Drosophila* ortholog of HIF1α – an interaction that was reciprocally identified in Sima/HIF1α interaction datasets. Structural modeling predicted binding between the dERR ligand-binding domain and a conserved LXXLL motif in Sima/HIF1α. Notably, the predicted dERR–Sima/HIF1α interaction interface includes residues previously shown to be essential for *in vitro* binding, providing independent support for this protein–protein interaction.

**Figure 1. The Drosophila Estrogen-Related Receptor (dERR) is predicted to bind Sima/HIF1α by FlyPredictome:** Predicted Aligned Error (PAE) heatmap of dERR and Sima/HIF1α interaction. (A) Each cell in the PAE heatmap represents the expected positional error between pairs of residues; low values (dark blue) indicate high confidence in their relative positioning. Off-diagonal blue regions between dERR and Sima/HIF1α indicate a confidently predicted interaction interface. Predictions are ranked from 1 to 5 according to confidence, with rank 1 representing the highest-confidence model. (B) Predicted interaction interface between dERR and Sima/HIF1α. The top bar represents the dERR protein sequence, and the bottom bar represents the Sima/HIF1α protein sequence. Light blue indicates the predicted interaction region in dERR, with dark blue highlighting contact residues. In Sima/HIF1α, the interaction region is shown in pink, with contact residues highlighted in red. (C) Predicted interacting amino acid sequences between dERR and Sima/HIF1α. Similar to (B), dark blue indicates the predicted interaction region in dERR, with contact residues highlighted in bold. Amino acid residues previously shown to influence dERR–Sima/HIF1α binding in yeast two-hybrid assays are marked by dark blue asterisks (Li et al., 2013). In Sima/HIF1α, the interaction region is shown in red, with contact residues similarly indicated in bold. The red box marks the LXXLL motif, and amino acid residues previously shown to influence dERR–Sima/HIF1α binding in yeast two-hybrid assays (Li et al., 2013) are marked by red asterisks. (D,E) Predicted 3D structures of the interaction interface between dERR and Sima/HIF1α. Amino acid residues are colored according to the same scheme as in (B). (D) Predicted contact residues within the dERR ligand binding domain (LBD) are noted with dark blue circles and labeled according to their positions in the protein. Amino acid residues whose substitution was previously shown to disrupt binding in yeast two-hybrid assays (Li et al., 2013) are marked by dark blue asterisks. (E) Similar to (D), the three leucine residues within the Sima/HIF1α LXXLL motif are highlighted with red circles and labeled according to their positions in the protein. Residues previously shown by yeast two-hybrid mutational analysis to be required for binding (Li et al., 2013) are marked by red asterisks.

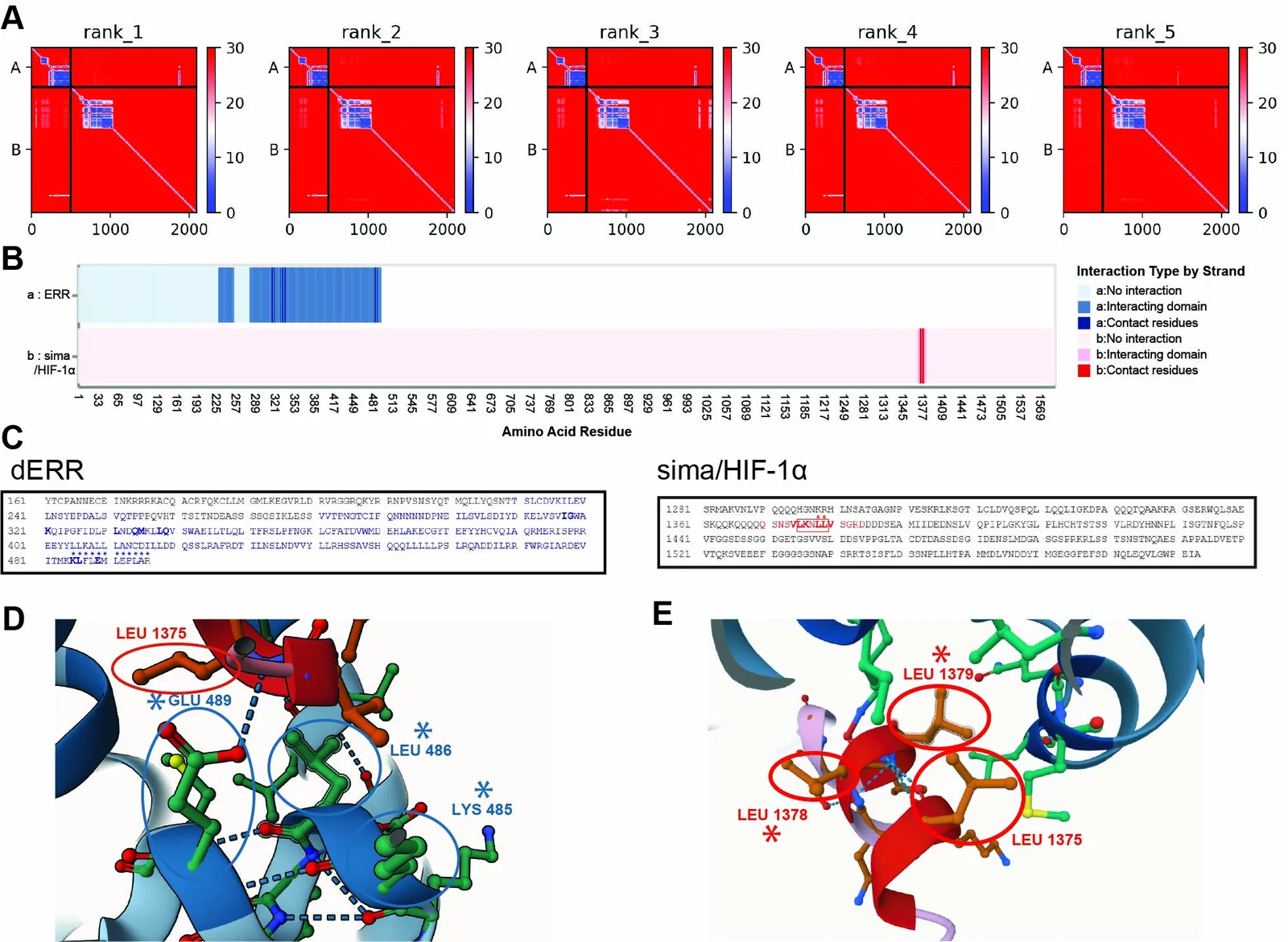



## Description

Nuclear receptors (NRs) constitute a large superfamily of transcription factors that regulate gene expression in response to hormonal, metabolic, and environmental cues (Jin et al., 2025, Taubenheim et al., 2021). These highly conserved proteins act as molecular sensors that translate extracellular and intracellular signals into coordinated transcriptional responses, thereby playing central roles in development, energy metabolism, reproduction, and physiological homeostasis across metazoans (Jin et al., 2025, Doering et al., 2022, Vanderhaeghen et al., 2022, Li et al., 2025, Love et al., 1983, Wu et al., 2023, Tao et al., 2020). Classical nuclear receptors are typically activated by endogenous small lipophilic ligands, including steroid hormones, thyroid hormones, and retinoids, which bind to the ligand-binding domain (LBD) and induce conformational changes that modulate DNA binding and recruitment of transcriptional cofactors (Jin et al., 2025, Tao et al., 2020).

In contrast, orphan nuclear receptors represent a subclass of the NR superfamily for which endogenous ligands have not been definitively identified (Jin et al., 2025). Rather than relying on ligand binding for activation, orphan nuclear receptors are frequently regulated through alternative mechanisms, including constitutive activity, post-translational modifications, and, importantly, protein–protein interactions with cofactors (Jin et al., 2025, Berger and Moller, 2002, Mohan and Heyman, 2003). Functionally, orphan nuclear receptors are involved in diverse biological processes, including metabolic regulation, developmental patterning, circadian rhythm control, and cellular stress responses (Jin et al., 2025, Safe et al., 2025, Yemanyi et al., 2023). Notably, these functions are highly conserved from invertebrates to mammals, underscoring their fundamental biological importance and evolutionary conservation (Jin et al., 2025, Yemanyi et al., 2023, Safe et al., 2025, Segraves, 1994, King-Jones and Thummel, 2005).

The *Drosophila* *melanogaster* Estrogen-Related Receptor (dERR) is the sole ortholog of the mammalian Estrogen-Related Receptor (ERR) family of orphan nuclear receptors (Tennessen et al., 2011, Fasteen et al., 2025, Zike et al., 2025). dERR has emerged as a critical regulator of metabolic reprogramming during development, particularly through its control of glycolytic metabolism during larval growth (Tennessen et al., 2011). Given its central role in metabolic regulation, identifying protein cofactors that interact with dERR is essential for understanding how its transcriptional activity is regulated in a context-specific manner. Moreover, elucidating these regulatory mechanisms has broader implications for human health, as ERR family members are widely implicated in metabolic diseases (Huss et al., 2015, Misra et al., 2016, Choi et al., 2022) and cancer (Ranhotra, 2015, Pradhan et al., 2026, Gulwani et al., 2023), where dysregulated energy metabolism is a hallmark phenotype (Hanahan and Weinberg, 2011). Thus, defining dERR-associated protein networks can provide mechanistic insight into conserved pathways, linking transcriptional regulation to metabolic homeostasis.

Despite its importance, systematic identification of nuclear receptor cofactors remains challenging. Traditional approaches, such as yeast two-hybrid screening, co-immunoprecipitation, and pull-down assays, have been widely used to detect protein–protein interactions (Rao et al., 2014). However, these methods are inherently low-throughput, labor-intensive, and often limited in their ability to capture transient or context-dependent interactions. In addition, they typically provide limited structural information regarding the interaction interface, which is critical for understanding the mechanistic basis of transcriptional regulation. These limitations highlight the need for complementary approaches that can efficiently predict and prioritize candidate interactions while also offering structural insights.

Recent advances in artificial intelligence (AI)–driven protein structure prediction have provided powerful new tools for addressing these challenges. FlyPredictome is a recently developed computational platform that leverages approximately 1.5 million pairwise predictions generated using AlphaFold-Multimer to infer protein–protein interactions in *Drosophila* (Kim et al., 2026). By integrating structural modeling with quantitative confidence metrics, including the interface Local Interaction Score (iLIS), FlyPredictome enables proteome-wide prediction and prioritization of candidate interactions. This structure-informed, high-throughput approach is particularly well suited for identifying previously unrecognized cofactors of orphan nuclear receptors.

To identify potential interacting partners of dERR, we queried the FlyPredictome database and ranked candidate proteins based on their iLIS values. Consistent with prior biochemical evidence, dERR itself emerged as the top-scoring interaction partner, reflecting its known ability to form homodimers through its ligand-binding domain (Vanacker et al., 1999, Greschik et al., 2002, Horard et al., 2004, Giguère, 2008; Table S1 **)** . This result provides internal validation of the predictive framework and supports the reliability of the platform in identifying biologically relevant interactions. Among the high-confidence candidates, Sima, the sole *Drosophila* ortholog of mammalian hypoxia-inducible factor 1α (HIF1α; Lavista-Llanos et al., 2002), was identified as a prominent interactor, with an iLIS best score of 0.600 that placed it within the top 1% of predicted interactions (Table S1). Notably, this prediction was reciprocal, as dERR also ranked among the top 1% of predicted interactors for Sima/HIF1α (Table S1). These results are consistent with a significant interaction between dERR and Sima/HIF1α.

Intriguingly, previous studies have demonstrated that Sima/HIF1α can physically interact with dERR (Li et al., 2013), and that *sima* mutant animals exhibit metabolic and developmental defects during embryonic and larval stages that closely resemble those observed in *dERR* mutants (Heidarian et al., 2025). The predicted dERR–Sima/HIF1α interaction is therefore consistent with a biologically relevant mechanism that may underlie the overlapping phenotypes observed in the two mutant backgrounds. To further characterize the predicted dERR–Sima/HIF1α interaction, we examined the structural interface generated by FlyPredictome ( Figure 1A –E). Interface mapping localized the interaction to the ligand-binding domain of dERR and a conserved LXXLL motif within Sima/HIF1α. Notably, LXXLL motifs are well-established nuclear receptor-binding motifs that interact with the activation function-2 (AF-2) surface of receptor ligand-binding domains (Savkur and Burris, 2004, Plevin et al., 2005). This binding arrangement is highly consistent with previous studies demonstrating a physical association between dERR and Sima/HIF1α (Li et al., 2013). Specifically, deletion of the C-terminal 12 amino acids of the dERR LBD ( Figure 1C ) abolishes Sima/HIF1α binding in vitro (Li et al., 2013), indicating that this region is required for complex formation. Likewise, substitution of the terminal leucine residues within the Sima/HIF1α LXXLL motif ( Figure 1C ) with alanines disrupts binding to dERR, demonstrating the functional importance of this motif (Li et al., 2013). Together, these findings show that the FlyPredictome model accurately recapitulates structural determinants previously demonstrated to mediate dERR–Sima/HIF1α binding, providing independent support for the biological relevance of the predicted complex.

In this study, FlyPredictome identified Sima/HIF1α as a high-confidence candidate interactor of dERR and predicted an interaction interface that recapitulates residues previously shown experimentally to be required for binding. These findings demonstrate how AI-based structural prediction can both discover candidate protein interactions and provide mechanistic insight into their molecular basis, thereby facilitating the identification and prioritization of biologically relevant regulatory networks. Several limitations of the FlyPredictome platform should nevertheless be considered. First, the predictive models do not account for post-translational modifications, such as phosphorylation, acetylation, or ubiquitination, which can influence protein conformation, stability, and interaction dynamics (Li et al., 2026, Lee et al., 2023). Second, the computational framework does not incorporate cellular or environmental context, including factors such as pH, compartmentalization, or molecular crowding, all of which can affect protein behavior in vivo (O'Brien et al., 2012, Fuentes-Lemus and Davies, 2023, Shaffer et al., 2005). Third, the predictions are limited to binary interactions and therefore do not capture the higher-order complexes that often mediate nuclear receptor function (Millard et al., 2013, Jin et al., 2025, Tao et al., 2020). Nevertheless, by recovering a previously documented dERR–Sima/HIF-1α interaction and predicting an interface that corresponds to experimentally implicated residues, FlyPredictome demonstrates the potential of AI-based structural approaches to generate mechanistic hypotheses and prioritize candidate regulatory interactions for future investigation. Among the predicted interaction partners shared by dERR and Sima/HIF-1α, Cyclin-dependent kinase 1 (Cdk1) emerged as a particularly intriguing candidate (Table S1). Cdk1 has previously been linked to metabolic regulation through phosphorylation of mitochondrial metabolic enzymes, thereby coordinating energy production with cell-cycle progression (Wang et al., 2014, Xie et al., 2019). The predicted interactions of Cdk1 with both dERR and Sima/HIF-1α therefore identify Cdk1 as a candidate component of this regulatory network and provide a testable hypothesis for future studies examining connections between metabolic, hypoxia-responsive, and cell-cycle regulation.

## Methods

The protein interactors of dERR and Sima/HIF1α were predicted using the FlyPredictome platform (Kim et al., 2026). The identifiers for dERR (FBgn0035849) and Sima/HIF1α (FBgn0266411) were obtained from FlyBase (release FB2026_02) (Öztürk-Çolak et al., 2024) and used as input for pairwise interaction analysis. FlyPredictome employs AlphaFold2-based (Jumper et al., 2021) multimer modeling to generate structural predictions of protein complexes and provide confidence metrics. Default thresholds for positive interactions were applied (iLIS > 0.223; LIS > 0.168; cLIS > 0.298; ipTM > 0.48; confidence score > 0.494).

The predicted dERR–Sima/HIF1α complex exhibited an interface local interaction score (iLIS) of 0.600 and an interface predicted TM-score (ipTM) of 0.55, indicating a high-confidence interaction. Multiple ranked models were generated, and the top-ranked model (rank 1 based on ipTM score) was selected for downstream analysis. Structural visualization was performed using a Mol*-based viewer (Sehnal et al., 2021), and the predicted complex was exported as a PDB file for further analysis and figure preparation.

## Data Availability

Description: FlyPredictome data for dERR and Sima/HIF1alpha. Table contains two sheets. First sheet is data from dERR analysis. Second sheet is data from Sima/HIF1alpha analysis.. Resource Type: Dataset. DOI: https://doi.org/10.22002/c9rn3-7dn93
